# The Impact of Diabetes Complications on the Physical Function of Maintenance Hemodialysis Patients

**DOI:** 10.7759/cureus.57867

**Published:** 2024-04-08

**Authors:** Shohei Minata, Genki Kudou, Shinsuke Imaoka

**Affiliations:** 1 Department of Rehabilitation, Oita Oka Hospital, Oita, JPN

**Keywords:** rehabilitation, skeletal muscle mass, physical functioning, diabetes mellitus, patients undergoing dialysis

## Abstract

This study investigated the impact of diabetes on the physical function of patients undergoing dialysis. This study included 22 patients undergoing outpatient dialysis with continued exercise therapy during dialysis at our hospital between January 2021 and August 2021. The participants were divided into two groups based on the presence or absence of diabetes, and various parameters were compared between the groups. To compare each physical function assessment and measurement of anterior thigh muscle thickness, repeated-measures analysis of variance was conducted to test for the presence of interactions and main effects.

Significant differences were observed in the absence of dyslipidemia (p < 0.01), high-density lipoprotein cholesterol level (p < 0.01), and foot sole skin perfusion pressure (p < 0.02). In terms of physical function, a main effect between the groups was observed in the five-time sit-to-stand test, and anterior thigh muscle thickness showed a main effect over time. Significant differences in the anterior thigh muscle thickness were observed between three and six months after the intervention (p < 0.05).

In patients undergoing dialysis with diabetes complications, a decrease in physical activity and lack of exercise can lead to a reduction in overall physical activity levels. Additionally, impairments such as peripheral neuropathy may contribute to an accelerated decrease in skeletal muscle mass.

## Introduction

The number of patients undergoing maintenance hemodialysis (patients undergoing dialysis) is increasing annually, with one in 358.9 people in Japan undergoing dialysis, according to national statistics [1]. According to the United States Renal Data System, the prevalence of patients undergoing dialysis in Japan is the second highest in the world after Taiwan [2]. The effectiveness of exercise therapy for patients undergoing dialysis has been established, with various reported benefits, including improved exercise tolerance, activities of daily living, quality of life, dialysis efficiency, and decreased mortality [3,4]. Particular attention has been focused on exercise therapy during dialysis, which effectively utilizes the time spent during the procedure. Yabe et al. reported significant improvements in physical function in older adult patients undergoing dialysis through a combination of aerobic exercise and strength training during a six-month dialysis period [5]. Performing exercise therapy during dialysis can lead to safe and continuous exercise therapy under medical supervision without the need for separate outpatient exercise visits.

Patients undergoing dialysis have a high incidence and prevalence of foot complications, which are associated with mortality [6]. Peripheral arterial diseases and comprehensive chronic lower limb ischemia (CLTI) are associated with peripheral neuropathy, sensory impairment, and infections, often posing amputation risk due to ischemia [7,8]. Additionally, there is a high incidence of complications, such as frailty and sarcopenia, leading to body movement difficulties, falls, and prolonged hospital stays [9]. Preventing a decline in physical function is crucial for maintaining a peaceful and independent daily life; however, the extent to which exercise therapy during dialysis can prevent a decline in physical function remains uncertain. The measurement of quadriceps muscle thickness using noninvasive ultrasound examinations has gained attention as a method for evaluating physical function [10]. Quadriceps muscle thickness correlates with the thigh cross-sectional area obtained from magnetic resonance imaging or computed tomography and is considered a useful indicator because it is related to the physical function of patients undergoing dialysis [11]. Recently, high diabetes incidence among patients undergoing dialysis with foot complications has been reported [1]. The presence of diabetes complications can lead to a decline in physical function due to muscle weakness and atrophy. However, studies investigating the progression of physical function in patients who continue exercise therapy during dialysis are limited. In this study, we investigated the impact of diabetes complications on the physical function of patients undergoing dialysis.

## Materials and methods

Study design

**Figure 1:**
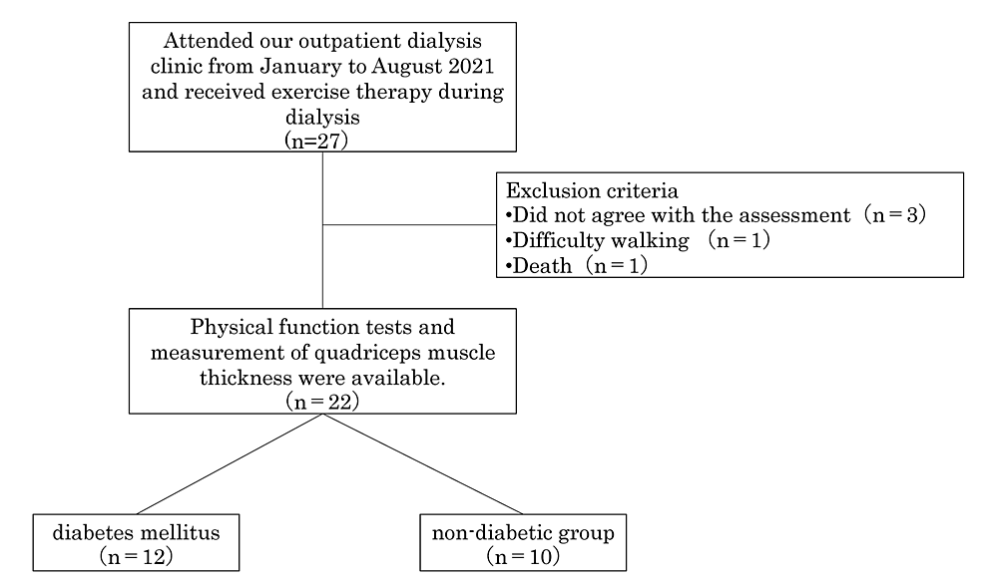
Flowchart of the participants

This study was a retrospective cohort study. The participants consisted of 22 patients who received outpatient dialysis at our hospital and were able to continue exercise therapy during dialysis between January 2021 and August 2021. Physical function and quadriceps muscle thickness were evaluated using ultrasonography at the initial assessment and then three and six months after the intervention (Figure 1). The exclusion criteria included individuals who did not consent to the evaluation (three participants), those who experienced difficulty walking during exercise (one participant), and those who passed away (one participant).

Assessment method

Baseline information included demographic data such as age, gender, height, weight, body mass index, years on dialysis, the presence of underlying diseases (hypertension, dyslipidemia, diabetes, heart failure, and disorders), and blood tests (total protein, albumin (alb), triglycerides, total cholesterol, low-density lipoprotein cholesterol, high-density lipoprotein (HDL) cholesterol, blood urea nitrogen, creatinine, estimated glomerular filtration rate, C-reactive protein, hemoglobin, sodium, chloride, potassium, calcium, and inorganic phosphorus). Vascular function tests, including the ankle-brachial pressure index, toe-brachial index, and skin perfusion pressure (SPP), were retrospectively investigated.

Physical function assessment

**Figure 2:**
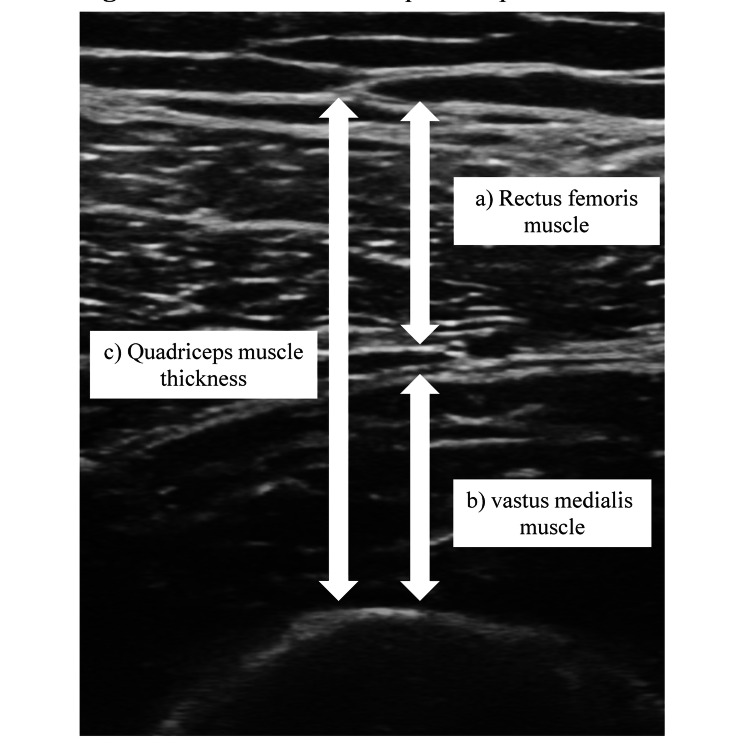
Measurement of quadriceps muscle thickness using an ultrasound imaging system ﻿﻿﻿(a) Rectus femoris muscle thickness. (b) Vastus medialis muscle thickness. (﻿﻿﻿c) Quadriceps muscle thickness (a + b).

Assessments of grip strength, the five-time sit-to-stand test, 10 m walking speed, and skeletal muscle mass were conducted, and the thickness of the quadriceps anterior muscle was measured. Grip strength was measured using a grip strength meter (Toei Light Grip Meter Grip D, Tokyo, Japan). Measurements were taken while the participant stood with the elbow joint extended and the forearm in mid-position. Grip strength was measured twice on each side, and the highest value was recorded. The five-time sit-to-stand test used a 40 cm high seat platform. Participants were instructed to stand up and sit down five times as quickly as possible with their arms crossed in front of their chest. The time to complete the task was measured. The 10 m walking speed test was conducted in the hallway in front of the dialysis room. Participants were instructed to walk 10 m at their maximum walking speed, and their time was recorded. Quadriceps muscle thickness was measured using an ultrasound imaging device (Venue 50A, ALCARE, Tokyo, Japan). Measurements were taken at the midpoint of the line connecting the greater trochanter and the lateral condyle of the femur, focusing on the vastus intermedius and rectus femoris. The ultrasound probe was adjusted perpendicular to the femur, and measurements were taken twice on each side, with the maximum value recorded (Figure 2). The measurements were conducted by a physiotherapist in the dialysis unit to ensure reproducibility, with the same individual performing all measurements to ensure consistency. Physical function assessments were performed every three months.

Exercise during dialysis

Exercise during dialysis included lower limb automatic movements, resistance training using weights (1.0-2.0 kg), and towel stretching in a supine position on the bed. The intervention began 60 minutes after the start of dialysis, and the exercise duration was set at approximately 10-20 minutes, following the standard exercise therapy protocol proposed by the Japanese Society of Nephrology Rehabilitation, with a frequency of three times per week.

Statistical analysis

The participants were categorized into two groups based on the presence or absence of diabetes. The normality of each variable was confirmed using the Shapiro-Wilk test. The χ2 test and Mann-Whitney U test were used for comparisons. Repeated-measures analysis of variance (ANOVA) was conducted to compare the measurement values of each physical function assessment and quadriceps anterior muscle thickness. The presence of interactions and main effects was tested. Mauchly's sphericity test was performed for repeated-measures ANOVA. If interactions and main effects were observed in the repeated-measures ANOVA, multiple comparison tests were conducted using Bonferroni’s method. Statistical analyses were performed using statistical software (SPSS version 26, IBM Corp., Armonk, NY), with the significance level set at 5%.

Ethical considerations

This study adhered to the Declaration of Helsinki and the ethical guidelines for medical research involving human participants. Data aggregation involved coding the patients’ names to ensure anonymity. The study objectives were verbally explained to the participants, and their consent was obtained. This study was approved by the Ethics Committee of Oita Oka Hospital (Approval Number: A0044). An opt-out approach was employed, and relevant information about the study was posted on the hospital's website.

## Results

Basic information of the participants is presented in Table 1.

**Table 1:** Basic information of the participants * P＜0.05. Mean ± standard deviation, median (25th-75th quartile range). BMI: body mass index; LDL: low-density lipoprotein; HDL: high-density lipoprotein; BUN: blood urea nitrogen; Cre: creatinine; eGFR: estimated glomerular filtration rate; CRP: C-reactive protein; Na: sodium; Cl: chloride; K: potassium; Ca: calcium; IP: inorganic phosphorus; ABI: ankle-brachial index; TBI: toe-brachial index; SPP: skin perfusion pressure.

	Diabetes group (n = 12)	Non-diabetic group (n = 10)	P-value
Age (years)	74.6±7.4	74.4±6.4	0.97
Male (example)	9	6	0.45
Height (cm)	158.9±8.6	158.5±13.4	0.73
Weight (kg)	56.8±7.0	55.9±14.5	0.91
BMI	22.5±2.7	22.0±4.2	0.68
Dialysis duration (years)	4.7 (2.0-8.7)	4.0 (2.0-6.0)	0.08
Presence of underlying disease			
Hypertension (%)	11 (91)	10	0.35
Lipid abnormalities (%)	9 (75)	1	0.01*
Cerebrovascular disease (%)	2 (16)	0	0.76
Heart failure (%)	5 (41)	4	0.93
Angina pectoris (%)	6 (50)	6	0.63
Blood test			
Total protein (g/dl)	6.1±0.5	6.6±0.2	0.22
Albumin (g/dl)	3.4±0.3	3.5±0.2	0.41
Triglyceride (mg/dl)	151.4±100.9	101.3±43.2	0.15
Total cholesterol (mg/dl)	133.5±25.6	140.3±26.9	0.45
LDL cholesterol (mg/dl)	74.8±24.2	74.7±22.1	0.87
HDL cholesterol (mg/dl)	36.3±8.0	49.3±5.3	0.01*
BUN (mg/dl)	49.3±13.3	50.6±12.5	0.67
Cre (mg/dl)	10.0±2.1	8.9±0.2	0.49
eGFR (mL/min/1.73m_2_)	4.4±1.2	4.9±1.3	0.41
CRP (mg/dl)	0.3 (0.08-2.0)	0.1 (0.06-0.5)	0.31
Hemoglobin (g/dl)	12.1±1.1	11.7±0.9	0.49
Blood sugar level (mg/dl)	127.6±25.1	125.8±35.6	0.79
Na (mEq/l)	138.2±3.0	139.0±2.4	0.28
Cl (mEq/l)	102.3±2.6	104.9±3.2	0.08
K (mEq/l)	4.5±0.7	4.5±0.5	0.62
Ca (mg/dl)	8.6±0.8	8.6±0.7	0.97
IP (mg/dl)	4.8±0.6	4.2±1.2	0.49
ABI, right lower limb	1.1±0.3	1.1±0.1	0.22
Left lower limb	1.1±0.3	1.0±0.2	0.15
TBI, right toe	0.6±0.2	0.7±0.1	0.86
Left toe	0.7±0.3	0.6±0.2	0.75
SPP, dorsal right foot (mmHg)	62.5 (53.3-94.8)	71.0 (54.3-79.5)	0.75
Right sole (mmHg)	63.0 (52.2-86.0)	70.5 (62.8-78.5)	0.66
Dorsal left foot (mmHg)	65.5 (41.7-89.0)	64.5 (53.8-70.8)	0.85
Left sole (mmHg)	55.0 (41.7-67.0)	72.5 (66.8-79.5)	0.02*

**Figure 3:**
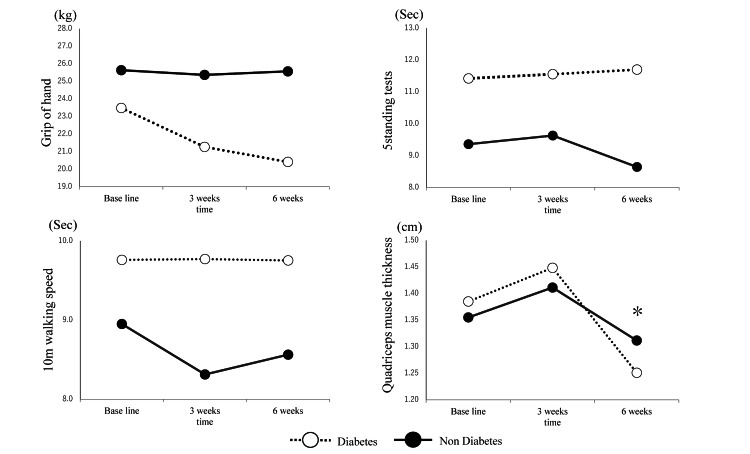
Changes in each physical function and quadriceps muscle thickness * P < 0.05: vs. three months.

In the two groups, i.e., diabetes and the non-diabetes groups, significant differences were observed in the presence of dyslipidemia (p < 0.01), HDL cholesterol (p < 0.01), and SPP at the plantar aspect of the foot (p < 0.02). Changes in physical function between the two groups are shown in Figure 3 and Table 2.

**Table 2:** Changes in physical function and quadriceps muscle thickness Mean ± standard deviation. * P < 0.05: vs. three months.

Item	Terms	Intervention	3 months	6 months		Two-way ANOVA	
					Time	Conditions	Reciprocal action
Grip strength	Diabetes mellitus	23.4±7.1	21.2±5.1	20.4±6.7	F = 3.171	F = 0.841	F = 2.689
	Non-diabetes mellitus	25.6±12.9	25.3±11.2	25.5±11.4	P = 0.054	P = 0.371	P = 0.082
5 standing tests	Diabetes mellitus	11.4±2.2	11.5±2.5	11.6±2.5	F = 0.787	F = 4.984	F = 1.696
	Non-diabetes mellitus	9.3±1.9	9.6±2.1	8.6±1.5	P = 0.464	P = 0.041	P = 0.201
10 m walking speed	Diabetes mellitus	9.7±1.8	9.7±1.9	9.7±1.5	F = 1.645	F = 2.114	F = 1.731
	Non-diabetes mellitus	8.9±1.5	8.3±1.3	8.5±1.9	P = 0.209	P = 0.165	P = 0.193
Quadriceps muscle thickness	Diabetes mellitus	1.38±0.3	1.44±0.4	1.25±0.4*	F = 8.300	F = 0.016	F = 0.289
	Non-diabetes mellitus	1.35±0.2	1.41±0.4	1.31±0.2*	P = 0.001	P = 0.900	P = 0.750

The five-time sit-to-stand test showed a significant main effect between the groups, and quadriceps muscle thickness demonstrated a significant main effect over time. However, no main effects or interactions were observed in the other parameters. Significant differences in quadriceps muscle thickness were observed between intervention months three and six (p < 0.05).

## Discussion

In this study, we investigated the impact of comorbid diabetes on the physical function of patients undergoing dialysis. The diabetes group showed a higher prevalence of dyslipidemia, lower HDL cholesterol levels, and a decrease in SPP than the non-diabetes group. Concerning physical function, the five-time sit-to-stand test scores were significantly lower in patients with diabetes comorbidities, and quadriceps muscle thickness demonstrated a main effect over time.

The association between dyslipidemia and prognosis in patients undergoing dialysis has been reported in several studies [12-14]. In particular, low HDL cholesterol levels are reported as a factor in the onset of acute myocardial infarction, with a significantly increased incidence reported for levels below 40 mg/dL [15]. A study investigating the effects of atorvastatin (20 mg/day) on 1,255 patients with type 2 diabetes undergoing hemodialysis reported an 8% reduction in the risk of cardiovascular death, nonfatal myocardial infarction, and cerebrovascular events, although the difference was not significant [16]. Therefore, a combined approach involving dietary guidance, exercise therapy, and careful consideration of the side effects and risks is crucial.

The prevalence of CLTI in patients undergoing dialysis is increasing annually, and early treatment, in addition to regular assessments, is crucial because of its poor prognosis. The SPP values in our study ranged from 50 to 70 mmHg, indicating a relatively high likelihood of wound healing. However, periodic vascular function tests are essential for preventing exacerbation, and practical guidance, such as foot care and walking instructions, is necessary [17].

Physical function in patients undergoing dialysis is influenced by factors such as inflammation, oxidative stress, and metabolic acidosis, which are associated with chronic kidney disease, aging, and comorbidities [18]. In a study by Ozdirenç et al., patients with comorbid diabetes had significantly lower values in grip strength, exercise tolerance, single-leg standing time, and trunk function than patients without diabetes [19]. In our study, no significant differences were observed in grip strength and 10 m walking speed; however, a significant difference was noted in the five-time sit-to-stand test.

Finally, regarding quadriceps muscle thickness, both the diabetes and non-diabetes groups showed a significant main effect over time. A previous study has reported that the quadriceps anterior muscle thickness in patients undergoing dialysis was 40.0 mm for men and women [20]. Our study demonstrated lower values than these findings, which were potentially accelerated by factors such as reduced physical activity, inactivity, and peripheral neuropathy in patients with comorbid diabetes.

One limitation of this study is that it was a retrospective observational study conducted in a single medical facility with a small number of participants. Additionally, information on activity levels, frequency of exercise therapy at home, and physical activity assessment were insufficient. Future research should aim to increase the number of participants and conduct a detailed investigation of the specific effects of diabetes comorbidity.

## Conclusions

In this study, we investigated the impact of diabetic complications on physical function in hemodialysis patients. It was shown that the diabetic group had significantly lower performance in the five-time sit-to-stand test and quadriceps muscle thickness compared to the non-diabetic group.

For hemodialysis patients with diabetic complications, it is crucial to consider the risk at an early stage of rehabilitation and provide exercise therapy with an appropriate load setting.
